# Letter and Speech Sound Association in Emerging Readers With Familial Risk of Dyslexia

**DOI:** 10.3389/fnhum.2018.00393

**Published:** 2018-10-02

**Authors:** Joanna Plewko, Katarzyna Chyl, Łukasz Bola, Magdalena Łuniewska, Agnieszka Dębska, Anna Banaszkiewicz, Marek Wypych, Artur Marchewka, Nienke van Atteveldt, Katarzyna Jednoróg

**Affiliations:** ^1^Laboratory of Psychophysiology, Nencki Institute of Experimental Biology, Polish Academy of Sciences (PAS), Warsaw, Poland; ^2^Institute of Psychology, Jagiellonian University, Krakow, Poland; ^3^Laboratory of Brain Imaging, Neurobiology Center, Nencki Institute of Experimental Biology, Polish Academy of Sciences (PAS), Warsaw, Poland; ^4^Faculty of Psychology, University of Warsaw, Warsaw, Poland; ^5^Department of Clinical Developmental Psychology & Institute LEARN!, Faculty of Behavioural and Movement Sciences, Vrije Universiteit Amsterdam, Amsterdam, Netherlands

**Keywords:** letter-speech sound association, audiovisual integration, dyslexia, reading fluency, familial risk

## Abstract

In alphabetic scripts, learning letter-sound (LS) association (i.e., letter knowledge) is a strong predictor of later reading skills. LS integration is related to left superior temporal cortex (STC) activity and its disruption was previously observed in dyslexia (DYS). Whether disruption in LS association is a cause of reading impairment or a consequence of decreased exposure to print remains unclear. Using fMRI, we compared activation for letters, speech sounds and LS association in emerging readers with (FHD+, *N* = 50) and without (FHD−, *N* = 35) familial history of DYS, out of whom 17 developed DYS 2 years later. Despite having similar reading skills, FHD+ and FHD− groups showed opposite pattern of activation in left STC: In FHD− children activation was higher for incongruent compared to congruent, whereas in FHD+ it was higher for congruent LS pairs. Higher activation to congruent LS pairs was also characteristic of future DYS. The magnitude of incongruency effect in left STC was positively related to early reading skills, but only in FHD− children and (retrospectively) in typical readers. We show that alterations in brain activity during LS association can be detected at very early stages of reading acquisition, suggesting their causal involvement in later reading impairments. Increased response of left STC to incongruent LS pairs in FHD− group might reflect an early stage of automatizing LS associations, where the brain responds actively to conflicting pairs. The absence of such response in FHD+ children could lead to failures in suppressing incongruent information during reading acquisition, which could result in future reading problems.

## Introduction

In alphabetic scripts, learning the association between letters and speech sounds (LS; i.e., letter knowledge) is a critical step in reading acquisition. LS knowledge is a strong predictor of later reading skills across many languages (Schatschneider et al., 2004; Caravolas et al., 2012). The pace of LS acquisition depends on a given script, especially its orthographic transparency, i.e., the degree of regularity in LS correspondence (Seymour et al., 2003). In transparent orthographies, most children master LS associations within 1 year of reading instruction and acquire reading effortlessly (Blomert and Vaessen, 2009). Although learning letter-sound (LS) associations happens at the very start of reading acquisition or already prior to reading acquisition, the full integration of LS pairs requires practice, and might take years to become fully automated.

However, around 10 percent of children struggle with reading acquisition and develop persistent reading difficulties, i.e., dyslexia (DYS; Shaywitz et al., 1998). The risk of developing DYS is substantially increased in children whose first-degree relatives had a history of reading problems (up to 30%–40% instead of 10% in general population, Snowling and Melby-Lervåg, 2016). According to a recent meta-analysis, children with family history of DYS (FHD+) face challenges in acquiring letter knowledge in preschool, which might result in later reading difficulties (Snowling and Melby-Lervåg, 2016).

Several fMRI studies examined brain response to LS association (congruent, where letters correctly denote speech sounds, and incongruent with non-matching LS pairs) in typical and reading disabled populations. In typically reading Dutch adults, response in the superior temporal cortex (STC) was enhanced by congruent and suppressed by incongruent LS pairs (van Atteveldt et al., 2004; Blau et al., 2009). Furthermore, adults with DYS underactivated STC, relative to typical readers, during congruent LS pairs processing (Blau et al., 2009). The decrease in activation was related to reduced processing of speech sounds, which in turn predicted the subjects’ phonological skills. Alterations in neural activity of the STC were also observed in 10-year-old Dutch children with DYS (Blau et al., 2010). While typical readers showed a strong congruency effect (higher activation to congruent than incongruent LS pairs), readers with DYS showed weaker congruency in left planum temporale/Heschl’s sulcus (PT/HS) and bilateral superior temporal sulcus (STS). The weaker congruency effect was further related to decreased LS matching knowledge and reading skills. Furthermore, in unisensory conditions, DYS readers compared to controls had lower activity in bilateral anterior STC for speech sounds and fusiform gyri (FG) for visual letters.

In less transparent orthographies, LS pairs induced a reversed congruency effect, namely stronger responses in the STC for incongruent compared to congruent grapheme-phoneme pairs in adult English typical readers (Holloway et al., 2015). Similarly, Swiss-German typical adolescent readers had enhanced brain activation to incongruent compared to congruent LS and consonant-vowel-consonant associations in left STC and FG, while the reversed pattern was observed in readers with DYS (Kronschnabel et al., 2014).

Although studies agree that LS integration as reflected by neural congruency effect is deficient in struggling readers across different alphabetic orthographies, the results are rather mixed in terms of the directionality of the congruency effect. The influence of orthographic transparency, stimulus properties (i.e., grain size) and developmental factors may contribute to this disparity. Moreover, since previous fMRI studies examined LS association in adults or children with at least 3 years of reading experience, it remains unclear whether the neural disruption in LS association is a cause of DYS or a consequence of decreased exposure to print. There is only one study (Nash et al., 2017) that addressed this issue by comparing the degree of LS integration between DYS readers and reading-matched controls. They did not find group differences, suggesting that the deficit is rather a consequence of the reading deficit than the cause.

We analyzed data from 85 Polish 7-year-old beginning readers with (FHD+) and without (FHD−) familial history of DYS, with similar early reading skills, out of whom 17 obtained DYS diagnosis 2 years later. If a different pattern of neural response for LS associations in left STC is inherent to reading deficits it should be already present at the beginning of literacy acquisition in FHD+ children especially those who later develop DYS. If however it is a consequence of impoverished reading experience FHD+ children should not differ from their FHD− peers in brain response to letters, speech sounds and LS pairs, as they still do not differ in reading experience at this stage of literacy acquisition.

## Materials and Methods

### Participants

We recruited 120 children from the last class of kindergarten and first grade of primary school for the purpose of longitudinal study on DYS. First graders had on average 3.62 months (SD = 2.01 range 1.20–7.80) of formal reading instruction. The results from other fMRI tasks on the same sample were described before (Dębska et al., 2016; Chyl et al., 2018). The inclusion criteria were: typical IQ (≥25th percentile in Raven’s Colored Progressive Matrices), birth at term (≥37 weeks), right-handedness, monolingualism (speaking Polish as their native language), normal (or corrected to normal) vision, normal hearing, no history of neurological illness or brain damage and no symptoms of ADHD. The study was approved by the Warsaw University Ethical Committee and all children and their parents gave informed consent to the study in accordance with the Declaration of Helsinki.

Due to excessive motion during fMRI scanning (*n* = 20), failing to complete two runs (*n* = 4) or dropping out from the study before DYS diagnosis (*n* = 11), 35 children were excluded from the current analyses. Specifically out of 109 children who participated in the longitudinal study until DYS diagnosis we had to deselect 24: nine FHD+ who developed DYS, eight FHD+ and seven FHD− who became typical readers. The final sample included 85 children: 35 FHD− (21 girls, 14 boys; mean age: 6.89 years (range: 5.93–8.04)) and 50 FHD+ (30 girls, 20 boys; mean age: 6.92 years (range: 5.52–8.06)). Children from the FHD+ group had at least one first degree relative with DYS diagnosis (65.6%), or at least one parent who scored greater than 40 points on the Adult Reading History Questionnaire (ARHQ, Lefly and Pennington, 2000) as specified in previous studies (Maurer et al., 2003; Black et al., 2012).

To control for non-verbal IQ, Raven’s Colored Progressive Matrices were used (Szustrowa and Jaworowska, 2003). Parental socioeconomic status (SES) was measured with Hollingshead’s (1975) index of social status based on parental education and profession; two families did not answer SES questionnaire. In case of four children fathers could not be contacted and thus their ARHQ scores could not be estimated. The two groups did not differ in age, sex, grade, IQ and parental SES (for details see Table 1).

**Table 1:** Demographic characteristics of FHD− and FHD+ participants, as well as dyslexic (DYS) and typical reading (TR) children together with group differences.

	FHD+	FHD−	Test statistics	DYS	TR	Test statistics
	*n* = 50	*n* = 35	FHD+ vs. FHD−	*n* = 17	*n* = 68	DYS vs. TR
Gender	20 B, 30 G	14 B, 21 G	*Chi*_(1)_ = 0 *p* = 1	8 B, 9 G	26 B, 42 G	*Chi*_(1)_ = 0.441, *p* = 0.507
Grade	12 K, 38 FG	9 K, 26 FG	*Chi*_(1)_ = 0.033, *p* = 0.857	6 K, 11 FG	14 K, 54 FG	*Chi*_(1)_ = 1.635, *p* = 0.202
Age (years)	6.92 (0.58)	6.89 (0.57)	*t*_(83)_ = −0.189, *p* = 0.851	6.74 (0.56)	6.96 (0.57)	*p*^b^ = 0.176
SES	46.86 (12.20)	50.17 (8.00)	*t*_(81)_ = 1.513, *p* = 0.134	42.63 (11.35)	49.74 (9.89)	*p*^b^ = 0.016*
ARHQ mother	37.66 (13.50)	22.14 (7.76)	*t*_(83)_ = −6.700, *p* < 0.001*	34.24 (16.85)	30.94 (12.58)	*p*^b^ = 0.322
ARHQ father	41.63 (14.30)	25.46 (7.21)	*t*_(79)_ = −6.641, *p* < 0.001*	37.67 (11.77)	33.94 (14.63)	*p*^b^ = 0.281
IQ (sten)	7.46 (1.42)	7.60 (1.24)	*t*_(83)_ = 0.471, *p* = 0.639	7.06 (1.59)	7.60 (1.25)	*p*^b^ = 0.106

Mean (SD) are depicted. B: boys; G: girls; K: kindergarten; FG: first grade of elementary school; ^b^ Bootstrap statistics. *marks significant effects.

Two years after the fMRI experiment, we conducted a formal diagnosis of DYS using a dedicated battery of tests (Bogdanowicz et al., 2009) that enabled retrospective selection of children with DYS. The battery consisted of 10 tests: four of them assessing reading, two assessing writing, three measuring phonological skills, and a measure of rapid automatized naming (RAN). Children who achieved low scores (3rd sten and lower, corresponding to 11.3 percentile) in at least two reading subtests (out of four: sight word reading, pseudo-word reading, text reading and lexical decision task) were identified as dyslexics. Based on these criteria 17 children from the current sample were diagnosed with DYS (*N* = 17, mean age = 6.74, nine girls, eight boys). Twelve belonged to the FHD+ group and five to the FHD− group. The remaining 68 children developed typical reading skills (TR group, mean age = 6.96, 38 FHD+, 42 girls, 26 boys). Thus, the proportion of dyslexic children was similar in the FHD− (16.7%) and FHD+ (24%) group (*Chi*_(1)_ = 1.21, *p* = 0.27) in the analyzed sample, because of large sample of FHD+ DYS children, who did not have usable fMRI data from the LS association task. However, the proportion of dyslexic children was significantly larger in the FHD+ (31.3%) than FHD− (11.9%) group for the total sample of 109 children (*Chi*_(1)_ = 5.37, *p* = 0.02), who took part in the longitudinal study. Thus, the prevalence of DYS in the current study is similar to the one reported in recent meta-analysis (Snowling and Melby-Lervåg, 2016). DYS and TR children did not differ in age, sex, grade, IQ or parental ARHQ, however TR children had higher parental SES (see Table 1).

### Behavioral Measures

Before the fMRI experiment (on average 46 days and no more than 4 months), all children underwent behavioral testing. The Decoding Test (Szczerbiński and Pelc-Pekała, 2013) was used to assess early reading and phonological skills. It included tasks of letter knowledge (upper and lower cases), sight word and pseudo-word reading (score: the number of correctly read words or pseudowords in a minute), phoneme elision (score: the number of items correctly solved in a minute), and phoneme analysis (score: the number of correctly solved items). Since psychometric norms were available only for first graders and our sample also included kindergartners, raw scores were used. Early print skills were measured with an orthographic awareness test where children had to choose the letter string, which exists in Polish (for instance DAG trigraph exists in Polish orthography, while DGA does not; Awramiuk and Krasowicz-Kupis, 2014). The outcome measure was the raw number of correctly assigned trigraphs. The passive vocabulary was tested with the Picture Vocabulary Test: comprehension (Haman et al., 2012), where a child is asked to select one of four images that corresponds to a specific word. The test had been standardized and normalized only for children from 2;0 to 6;11 years, therefore raw scores were used in the analyses. RAN was measured with subtests objects and colors naming (Fecenec et al., 2013). The outcome measure was the average time (in seconds) needed to name all stimuli in two subtests.

A formal diagnosis of DYS was conducted using a standardized battery of tests (Bogdanowicz et al., 2009) and children who achieved low scores (equal or lower than the 3rd sten) in at least two reading subtests (out of four: sight word reading, pseudo-word reading, text reading and lexical decision task) were identified as DYS.

To investigate behavioral performance differences between the FHD+ and FHD− groups independent sample *t*-tests were used. Because of the unequal group sizes, to test which behavioral variables significantly differ between DYS and TR, we performed bootstrap analysis. First, for each variable, the actual between-group difference was calculated. The values from both groups were put together to one dataset. Next, from this dataset, two subsets with sizes equal to the sizes of actual groups (for e.g., *N*_(DYS)_ = 17, *N*_(TR)_ = 68) were generated by drawing with replacement, and the difference between the means of the subsets was calculated. This step was repeated 10,000 times and histograms represent the distributions of the obtained mean differences. We calculated the number of occurrences when absolute values of differences from the distribution exceeded the absolute value of the real between-group difference. Two-tailed *p*-value was estimated by dividing the obtained number by the number of drawings (i.e., 10,000).

### fMRI Task

The experiment consisted of two runs, each run having 12 stimulation blocks and 12 fixation periods. One block (15.6 s) consisted of three mini-blocks (5.2 s) and contained 12 stimuli (four per mini-block) and was repeated twice per run, resulting with 48 stimuli per condition. The order of blocks was pseudorandomized so that two blocks of the same kind were not displayed in a row. The procedure was adapted from van Atteveldt et al. (2004). In each block stimuli from one of six conditions were presented using Presentation software (Neurobehavioral Systems). There were four experimental conditions: unisensory visual letters and speech sounds corresponding to selected Polish single letters (consonants: B, C, D, G, H, J, K, L, M, N, P, R, S, T, W, Z; and vowels: A, E, I, O, U), multisensory congruent and incongruent LS pairs, as well as two control conditions: symbols (Greek letters unknown to children) and speech sounds transformed into noise-vocoded speech with an in-house script in Praat (Boersma and Weenink, 2001). This study focuses only on the four experimental conditions, and comparisons with control conditions will be presented in a separate publication. Children were instructed to pay attention to the stimuli very carefully. To ensure that children attended to the stimuli, we followed the procedure as in Blau et al. (2010). A line drawing of cat, a voice (saying “cat”) in the unisensory blocks, or a combination of the two in the multisensory blocks, was presented once per block (pseudo-randomized). Children were asked to press a button on a response-pad with left thumb every time they detect such stimuli.

### fMRI Data Acquisition

All participants were familiarized with the MRI environment and procedure in a mock scanner before the beginning of experimental session in the 3T Siemens Trio MR system (Siemens AG, Munich, Germany). We used sparse design sequence so that the stimuli could be presented during silent delay of volume acquisition, which minimized the effects of scanning noise on experimental activation (van Atteveldt et al., 2004). Functional MRI data were acquired using a T2* - sensitive, gradient echo planar imaging sequence covering the whole-brain (29 slices, slice thickness: 4 mm, 3 × 3 in-plane resolution, TR = 5.2 s (1.5 s of volume acquisition followed by 3.7 s delay), TE = 25 ms, matrix size: 64 × 64). The task was presented in two fMRI runs, each lasting for 6 min and 17 s (73 volumes), which in total gave 12 min and 34 s (146 volumes). Anatomical data were acquired using a T1 weighted sequence (176 slices, slice-thickness 1 mm, TR = 2.53 s, TE = 3.32 ms, flip angle = 7°, matrix size: 256 × 256, voxel size 1 × 1 × 1 mm).

### fMRI Data Preprocessing

The imaging data were analyzed with BrainVoyager QX 2.2.0 (Brain Innovation, Maastricht, Netherlands; Goebel et al., 2006). Functional data were preprocessed to correct for 3D motion artifacts (trilinear interpolation), linear drifts and low-frequency non-linear drifts (high pass filter “3 cycles/time course). All functional images were co-registered to the anatomical image. The anatomical image was then transformed into Talairach stereotaxic space (Talairach and Tournoux, 1988), and this transformation was applied to the aligned functional data. The functional images were spatially smoothed with a 6-mm FWHM Gaussian kernel. Finally, ART toolbox^1^ was used to detect motion-affected functional volumes (thresholds were adapted from Raschle et al. (2012): movement threshold: 3 mm, rotation threshold: 0.05 mm). If number of motion-affected volumes was higher than 20%, the participant was excluded from analysis.

### MRI Whole Brain Statistical Analyses

Both experimental and control conditions were modeled in single subject design matrix together with motion parameters and separate regressors for each volume that was identified as motion-affected by ART toolbox. Second level statistical analyses were adapted from Blau et al. (2010). Second level analyses were performed using the general linear model (GLM) approach. The first analysis was a single factor model including four experimental conditions (i.e., letters, speech sounds, congruent LS pairs and incongruent LS pairs) as separate predictors, and was used to determine brain regions involved during the experimental tasks for the whole sample. The statistical map from this analysis (all four experimental conditions vs. baseline (rest period) contrast) was used as a mask (thresholded at *p* = 0.05) for subsequent GLMs. Next, two separate GLMs (GLM1 and GLM2) were computed for FHD− and FHD+ children, to evaluate the spatial pattern of activation for letters and speech sounds in each group separately (corrected for multiple comparisons using false-discovery rate, *q*_(FDR)_ < 0.01).

Direct between-group comparisons for unisensory conditions-letters and speech sounds were performed in GLM3. GLM 4 was a 2 × 2 factorial model including FHD status and multimodal conditions: congruent and incongruent pairs of letters and speech sounds. The congruency effect: difference between congruent and incongruent letter-speech sound pair calculated in the GLM4 was used to identify multisensory integration sites (Van Atteveldt et al., 2007). We applied the same statistical threshold as in the previous study on DYS children (Blau et al., 2010), i.e., voxel-wise threshold of *p* < 0.01, corrected for multiple comparisons using cluster extent threshold of *p* < 0.05 (Forman et al., 1995; Goebel et al., 2006). The clusters are reported in the Talairach space and displayed on average brain from all participants. Additionally, in Supplementary Table S1 we report the results of whole brain analyses with a more stringent voxel-wise threshold of *p* < 0.005 with cluster extent threshold of 50 voxels similarly to other pediatric fMRI studies (e.g., Raschle et al., 2012; Wang et al., 2018; Yu et al., 2018).

### fMRI ROI Analyses

To further explore the differences between the groups in unisensory and multisensory conditions in regions previously reported to differ between dyslexic and control subjects, ROI analyses were performed. Seven ROIs: left and right FG (for letter condition), left and right anterior superior temporal gyi (aSTG; for speech sound condition) as well as left and right STS and left planum PT/HS (for multisensory conditions) were examined by creating a 4 mm spheres around the peak coordinates taken from Blau et al. (2010). The percent signal change in these ROIs was compared between FHD+ and FHD− children. The statistical threshold was corrected for the number of ROIs with *p* < 0.025 for letters and speech sounds, and *p* < 0.016 for multisensory conditions.

Next, similarly as for behavioral variables, we retrospectively explored brain activity differences between DYS and TR groups by the means of bootstrap analysis in ROIs taken from Blau et al. (2010) as well as in regions showing significant differences between FHD− and FHD+ children in the whole brain analysis.

Moreover, we calculated Pearson’s correlations between scores on reading related tests (word reading, orthographic awareness, phoneme analysis and elision) and the strength of the fMRI congruency effect. The correlations were performed in regions showing a significant group × congruency interaction in the current study and in (Blau et al. (2010); i.e., left and right STS and left PT/HS) in FHD+ and FHD− children, and in TR and DYS groups separately. The statistical threshold was corrected for the number of ROIs and behavioral measures (*p* < 0.007).

Finally, in the sample of first graders, we computed correlations between time of reading instruction, behavioral performance and congruency effects in ROIs taken from the whole brain analysis of FHD status and from Blau et al. (2010). Further to examine if the same pattern of results in present for beginning readers and prereaders, we performed additional ROI analyses reported in Supplementary Materials.

## Results

### Behavioral Results

FHD+ children did not differ significantly from FHD− group with respect to performance on early reading, phonological awareness or orthographic awareness tests (for details see Table 2).

**Table 2:** The results from behavioral tests in FHD+ and FHD− groups, as well as DYS and TR groups.

	FHD+	FHD−	Test statistics	DYS		Test statistics
	*n* = 50	*n* = 35	FHD+ vs. FHD−	*n* = 17	DYS vs. TR
Letter knowledge (upper and lower case, max. 64)	46.92 ± 18.41 (0–64)	47.40 ± 18.07 (0–64)	*t*_(83)_ = 0.119, *p* = 0.905	31.76 ± 20.00 (0–59)	51.26 ± 15.28 (4–64)	*p*^b^ < 0.001*
Word read per minute	15.18 ± 15.96 (0–52)	18.43 ± 21.01 (0–69)	*t*_(83)_ = 0.81, *p* = 0.420	4.65 ± 5.13 (0–16)	19.69 ± 18.84 (0–69)	*p*^b^ = 0.003*
Pseudowords read per minute	13.97 ± 13.40 (0–47)	12.96 ± 13.61 (0–40)	*t*_(82)_ = 0.336, *p* = 0.738	4.00 ± 5.03 (0–15)	15.90 ± 13.74 (0–47)	*p*^b^ < 0.001*
Phoneme analysis (solved items, max. 12)	6.60 ± 4.44 (0–12)	7.77 ± 4.05 (0–12)	*t*_(83)_ = 1.241, *p* = 0.218	3.59 ± 3.48 (0–10)	8.04 ± 4.02 (0–12)	*p*^b^ < 0.001*
Phoneme elision (solved items per minute)	4.10 ± 4.23 (0–13)	4.26 ± 4.52 (0–15)	*t*_(83)_ = 0.164, *p* = 0.870	1.47 ± 2.28 (0–7)	4.88 ± 4.42 (0–15)	*p*^b^ = 0.004*
RAN object and color subtests (seconds)	127.04 ± 29.49 (77–203)	135.43 ± 35.41 (91–268)	*t*_(83)_ = 1.188, *p* = 0.238	152.12 ± 40.69 (113–268)	125.16 ± 26.66 (77–196)	*p*^b^ = 0.003*
Orthographic awareness (solved items, max. 30)	18.45 ± 4.74 (8–29)	20.06 ± 4.42 (13–29)	*t*_(83)_ = 1.563, *p* = 0.122	15.38 ± 3.92 (8–25)	20.03 ± 4.31 (11–29)	*p*^b^ < 0.001*
Vocabulary (max. 88)	79.48 ± 5.40 (64–87)	77.20 ± 7.29 (57–88)	*t*_(83)_ = −1.657, *p* = 0.101	76.59 ± 6.14 (64–85)	79.25 ± 6.04 (57–88)	*p*^b^ = 0.145
Sight word reading* (sten)	5.82 ± 2.04 (1–10)	6.09 ± 1.98 (2–10)	*t*_(83)_ = 0.598, *p* = 0.552	3.65 ± 1.50 (1–6)	6.51 ± 1.69 (4–10)	*p*^b^ < 0.001*
Pseudo-word reading* (sten)	4.94 ± 1.89 (1–10)	5.24 ± 1.69 (1–8)	*t*_(83)_ = 0.734, *p* = 0.465	2.88 ± 1.41 (1–6)	5.61 ± 1.45 (3–10)	*p*^b^ < 0.001*
Text reading* (sten)	4.63 ± 2.08 (1–9)	5.27 ± 2.60 (1–10)	*t*_(81)_ = 1.235, *p* = 0.221	2.12 ± 0.86 (1–3)	5.62 ± 1.99 (2–10)	*p*^b^ < 0.001*
Lexical decision task* (sten)	5.16 ± 2.21 (1–10)	5.91 ± 2.17 (2–10)	*t*_(83)_ = 1.544, *p* = 0.126	2.41 ± 0.87 (1–4)	6.24 ± 1.72 (3–10)	*p*^b^ < 0.001*

Means ± SD together with range in parentheses are presented. *Battery of tests used for dyslexia diagnosis; ^b^ bootstrap statistics.

The bootstrap analyses revealed that at the beginning of reading acquisition, children who were 2 years later classified as DYS, had lower scores in letter knowledge, word and pseudoword reading, phoneme analysis, elision, RAN and orthographic awareness than TR children (for detailed scores see Table 2). No significant differences were found in passive vocabulary between DYS and TR children.

### fMRI Results

#### Whole Brain Analyses

**Figure 1:**
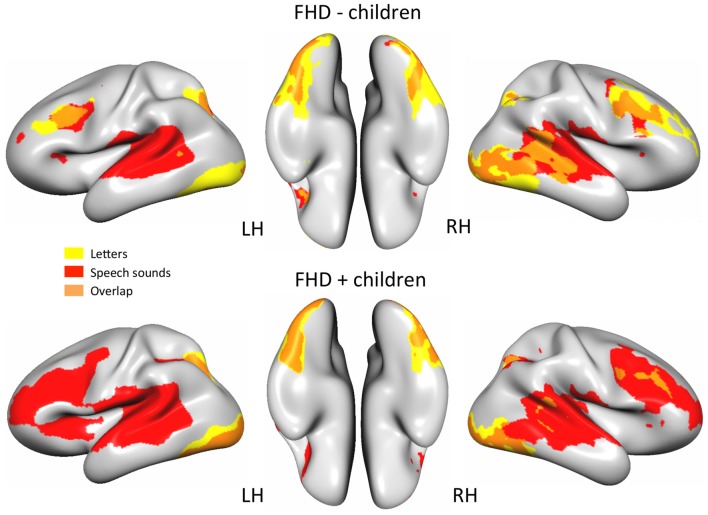
Brain areas involved in processing letters (yellow), speech sounds (red) or both unisensory conditions (orange) in children without (FHD−) and with (FHD+) family history of dyslexia (DYS; *q*_(FDR)_ < 0.01).

Figure 1 depicts brain activity in FHD− and FHD+ children in response to unisensory presented letters and speech sounds as well as the overlap of brain activity for both conditions (GLMs 1 and 2).

**Figure 2:**
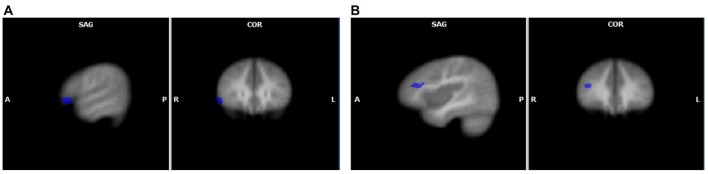
Unisensory group effects for speech sounds with increased activation in FHD+ compared to FHD− children in the right inferior frontal gyrus **(A)** and in the right middle frontal gyrus **(B)**. The clusters are displayed on average brain from all participants at voxel-wise threshold of *p* < 0.01, corrected for multiple comparisons using cluster extent threshold of *p* < 0.05.

When the two groups were directly compared for each unisensory condition (GLM3) significant differences in brain activity were found only for speech sounds. FHD+ children had higher activity for speech sounds than their FHD− peers in right middle and inferior frontal gyri (see Table 3 and Figure 2). No significant differences between the groups were found for letter processing.

**Table 3:** Group differences in response to speech sounds and interaction between group and multisensory conditions.

Brain region	BA	Hemisphere	*x*	*y*	*z*	t/F	Voxels
**Speech sounds: FHD+ > FHD−**							
Inferior Frontal Gyrus	10	R	54	39	−2	−4.01	910
Middle Frontal Gyrus	46	R	39	32	22	−3.20	715
**Congruency effect x FHD group interaction**							
Inferior Temporal Gyrus	37	R	54	−49	−23	17.62	648
Planum Temporale, Superior temporal gyrus	41/13	L	−42	−16	19	13.77	1700

*Note: The results are reported at voxel-wise threshold of *p* < 0.01, corrected for multiple comparisons using cluster extent threshold of p < 0.05*.

**Figure 3:**
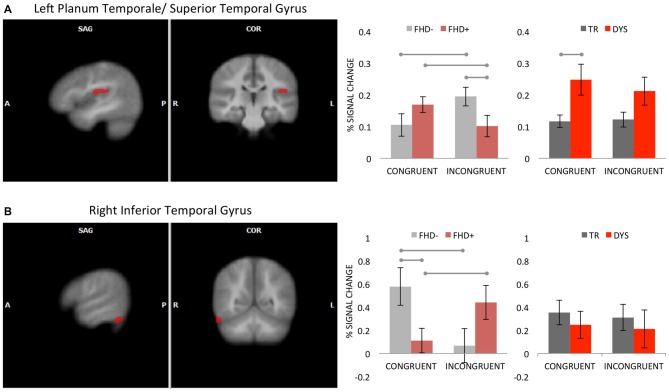
Interaction effect between group and multisensory conditions (congruent, incongruent) in the left planum temporale/superior temporal gyrus (PT/STG) **(A)** and in the right inferior temporal gyrus **(B)**. The clusters are displayed on average brain from all participants at voxel-wise threshold of *p* < 0.01, corrected for multiple comparisons using cluster extent threshold of *p* < 0.05. Bar graphs illustrate the percent signal change with SEM for multisensory conditions in FHD− and FHD+ (horizontal lines illustrate significant *post hoc* tests) as well as in DYS and typical reading (TR) children (horizontal line illustrates significant bootstrap statistics).

A significant interaction between group and multisensory conditions (GLM4) was found in the left PT/STG and right inferior temporal gyrus (ITG, for details see Table 3 and Figure 3). In the left PT/STG it was driven by higher activation to incongruent, relative to congruent LS pairs in FHD− (*p* = 0.021), and a reversed pattern (higher activity for congruent pairs) in FHD+ children (*p* = 0.037). The two groups differed for incongruent (FHD− > FHD+; *p* = 0.029), but not for congruent condition. In the right ITG the pattern was opposite, namely the activation was higher for congruent, relative to incongruent LS pairs in FHD− children (*p* = 0.004), whereas a reversed effect (higher activity for incongruent pairs) was present in FHD+ children (*p* = 0.024). In this cluster the groups differed for congruent (FHD− > FHD+; *p* = 0.008), but not for incongruent condition.

#### ROI Analyses

Further analysis of seven ROIs based on regions distinguishing between DYS and control children in Blau et al. (2010) revealed a trend for lower activation in FHD+ compared to FHD− children in the left fusiform gyrus for letter processing (*x* = −36, *y* = −51 *z* = −17; *t* = 1.95, *p* = 0.056). In the left PT/HS (*x* = −42, *y* = −28, *z* = 13) we found significant interaction between group and multisensory conditions (*F*_(1,83)_ = 6.22, *p* = 0.012). The groups differed only in the incongruent condition (*p* = 0.009), where FHD− children had higher activation than FHD+ children. Additionally, FHD+ children presented higher activation for congruent compared to incongruent LS pairs (*p* = 0.029), while no differences between the conditions were observed in FHD− children. We did not find any FHD effects in the other ROIs.

#### Bootstrap Analyses (Comparisons Between TR And DYS Groups)

**Figure 4:**
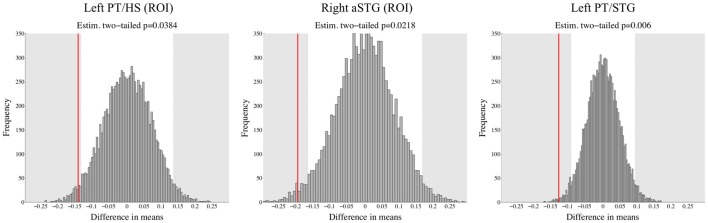
Results obtained using bootstrap analysis based on percent signal change data in two ROIs from Blau et al. (2010) and in the left PT/STG cluster from the whole brain analysis of FHD+ and FHD− children. Red line represents the actual observed value of difference in means between TR and DYS children, whereas shaded gray areas depict significant differences (two-tailed *p* < 0.05). DYS compared to TR showed higher brain response to congruent letter-sound (LS) pairs in left PT/HS and in left PT/STG as well as higher brain response to speech sounds in right anterior STG (aSTG).

For ROIs taken from Blau et al. (2010), a trend for differences appeared in left PT/HS: DYS children had higher activity than TR group for congruent LS pairs (*p* = 0.039). Additionally, in the right aSTG in response to speech sounds DYS group had significantly higher activity than TR group (*p* = 0.022). We did not find any DYS effects in the other ROIs taken from Blau et al. (2010). Furthermore, in the left PT/STG, an ROI showing significant interaction between FHD status and congruency in the whole brain analysis, DYS children had significantly higher activity than TR group for congruent LS pairs (*p* = 0.006). Figure 4 presents histogram distributions from bootstrap analysis together with the actual between group differences in percent signal change in these three brain regions.

#### Correlations With Behavioral Variables

**Figure 5:**
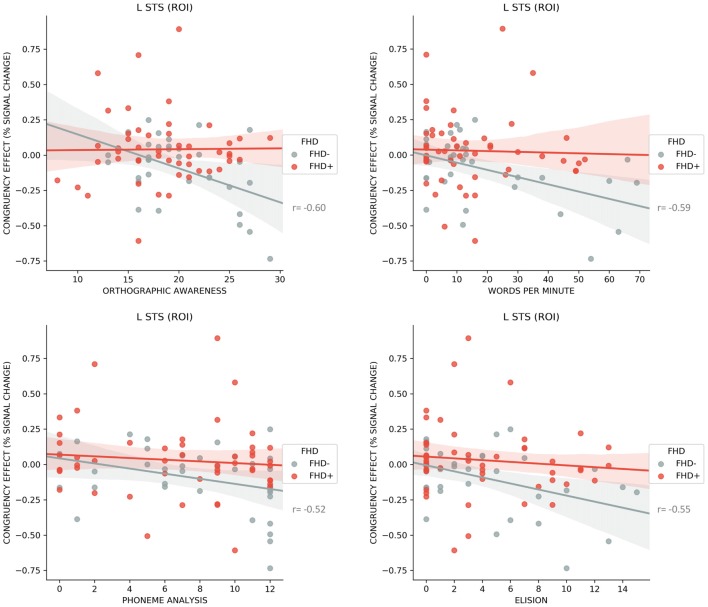
Correlations for FHD− and FHD+ children between the neural response to congruent vs. incongruent letter speech sound pairs (fMRI congruency effect) in left superior temporal sulcus (STS) and orthographic awareness, word reading, phoneme analysis and elision along with 95% confidence intervals. Correlations are significant only in FHD− children.

Several significant negative correlations between congruency effect (i.e., higher response for congruent compared to incongruent LS pairs) in the left STS ROI and early reading skills were found in FHD− children (word reading *r* = −0.59, *p* < 0.001; orthographic awareness *r* = −0.60, *p* < 0.001; phoneme analysis *r* = −0.52, *p* = 0.001; phoneme elision *r* = −0.55, *p* = 0.001; see Figure 5). None of the above correlations were significant in FHD+ group. We did not find significant correlations for the clusters showing significant FHD effects on the whole brain level.

When the sample was retrospectively split into TR and DYS, we found negative correlations in TR between congruency effect in the left STS and word reading (*r* = −0.34, *p* = 0.005), while correlations with phoneme analysis (*r* = −0.30, *p* = 0.012) and elision (*r* = −0.27, *p* = 0.027) did not survive the correction for multiple comparisons. None of the correlations were significant in the DYS group.

To test the relation between reading instruction, behavioral performance and brain activity, we correlated months of reading instruction that the first-grade children (*n* = 66) received with congruency effects in ROIs taken from the whole brain analysis of FHD status and from Blau et al. (2010). These correlations were performed in the whole sample of first graders and separately in FHD− (*n* = 28), FHD+ (*n* = 38) and TR children (*n* = 56), but not in DYS (*n* = 10) because of too few subjects. Importantly, no differences were found in months of reading instruction between FHD− and FHD+ and between TR and DYS children. The time of reading instruction was weakly positively correlated with word and pseudoword reading in the whole sample (*r* = 0.25, *p* = 0.047 and *r* = 0.32, *p* = 0.009), in FHD+ children (*r* = 0.33, *p* = 0.04 and *r* = 0.36, *p* = 0.025) and in TR (only pseudoword reading, *r* = 0.34, *p* = 0.011), however these correlations did not survive the correction for multiple comparisons (due to repeating the correlations for seven behavioral measures). On the neural level only in the whole sample of first graders and in FHD− children a weak negative correlation was found between months of reading instruction and congruency effect in right STS (*r* = −0.31, *p* = 0.012 and *r* = −0.46, *p* = 0.015 for the whole sample and FHD− first graders respectively). Again, these correlations were not significant after correction for multiple comparisons (due to repeating the correlations in five ROIs).

## Discussion

We examined brain response to letters, speech-sounds and LS pairs in emerging readers with and without familial risk for DYS and retrospectively assessed which of the observed effects are present in children who developed DYS 2 years later. Even though behaviorally FHD+ and FHD− groups did not differ with respect to early reading, phonological awareness and orthographic skills (similarly as in Specht et al., 2009 and characteristic of transparent orthographies) and the prevalence of DYS was also similar between the FHD groups in children qualified for the current analyses (see “Participants” section for details), we found brain activation differences for both unisensory and multisensory conditions in regions previously implicated in DYS. Children who later developed DYS compared to typical readers presented lower early reading skills and altered brain response in STC to speech sounds and congruent LS pairs.

In details, for multisensory conditions, we found an interaction between FHD group and LS congruency (congruent vs. incongruent LS pairs) in left STC and in right inferior temporal cortex. The cluster in left STC was in close proximity to the left PT/HS cluster where weaker congruency effect in DYS children was found previously (Blau et al., 2010). Curiously, in the current study the congruency effect in the left STC was of a different direction, i.e., FHD− children had higher brain response to incongruent compared to congruent LS pairs, while FHD+ children had the opposite pattern (higher brain activity for congruent compared to incongruent condition). The reversed direction of congruency effect was further confirmed in the ROI analysis, where the activity in the left PT/HS for incongruent condition was significantly higher in the FHD− than FHD+ group, while no group differences were found for the congruent condition. Additionally, children who developed DYS had significantly higher response than the typically reading group in the left STC (left PT/STG and left PT/HS ROIs) for congruent LS pairs. Finally, stronger response to the incongruent LS pairs (relative to congruent pairs, i.e., incongruency effect) in left STS ROI was positively related to early reading performance in FHD− children and (retrospectively in) typical readers. The congruency effect was not related to performance measures neither in FHD+ nor in DYS children. However, lack of correlation in DYS group could be explained by both smaller sample and more restricted range of behavioral performance in the lower end of the continuum.

In left PT/STG and left PT/HS, we observed group differences related to DYS or risk of DYS similarly as in previous studies on adult (STG, Blau et al., 2009) and older children (PT/HS, Blau et al., 2010). Next, we found that the congruency effect is negatively related to reading and reading related performance in typical readers and children without the risk of DYS in left STS but not PT/STG or PT/HS. Only in Blau et al. (2010) brain-behavior correlations were performed and even though for the whole sample of children significant relations with congruency effect in left STS and PT/HS were found, they were driven by group differences and became non-significant when the group factor was controlled for. PT/HS and surrounding STG are sensitive to acoustic features, and the former does not distinguish speech and non-speech (Price, 2012). STS on the other hand is more involved in speech than nonspeech, shows neural adaptation effects to phonological level information (Vaden et al., 2010), while bilateral lesion of STS often associated with word deafness (Stefanatos, 2008). Activity in left STS in response to both print and speech is also related to reading abilities in emerging readers (Chyl et al., 2018). That is why enhanced activation to congruent vs. incongruent LS in left HS and PT was putatively attributed to feedback from STS and STG to primary auditory cortex (van Atteveldt et al., 2004). Perhaps in typical beginning readers the more efficient the reading skills the more effective feedback from STS to auditory cortex, resulting in higher incongruency effect as compared to children at risk for DYS.

There might be several explanations for the reversed congruency effects in the left STC observed in the current study. First of all, the effect could be driven by differences in orthographic transparency. The observed direction of congruency effect of Polish children is more comparable to results obtained from English and Swiss-German (Kronschnabel et al., 2014; Holloway et al., 2015), where in typical readers higher brain response to incongruent compared to congruent stimuli was recorded. One could argue that it is specific for irregular orthographies with high LS mapping inconsistency. Indeed, one comparative study reported the highest inconsistency in English, followed by German, while Dutch was on the other end of LS ambiguity (Polish was not included, Borgwaldt et al., 2005). In this study transparency measurements were performed for single LS as well as letter clusters (rimes and onsets), thereby modeling knowledge of advanced readers. More recently (Schüppert et al., 2017) similar approach, based only on single LS correspondences (modeling beginning readers) was used in 16 European languages, including Polish. English was the least predictable, while Dutch had higher inconsistency for reading than German or Polish. Thus, the argument for orthography irregularity driving incongruency effect would not hold for beginning readers.

On the other hand, developmental, reading skill or even effects related to processing effort might modulate directionality of congruency effect as the currently examined sample is much younger and has less reading experience than all previously studied samples. The observed pattern could reflect an early stage in the process of LS integration in FHD− group, where the brain responds actively to the conflicting pairs. Only after automation, incongruent pairs could be suppressed. FHD+ on the other hand do not show the increased activation to conflicting pairs, but instead higher activity to congruent ones (especially those children who later develop DYS), which could later lead to failures in suppressing the incongruent information. This explanation would be consistent with studies showing that the automation in LS integration develops relatively slowly. For instance, reaction times of LS discrimination decisions steadily decreased during the whole range of Dutch primary school reading instruction (Froyen et al., 2009). This extended development towards automatic LS integration has been also observed in studies measuring EEG responses in a passive cross-modal “oddball” paradigm (Froyen et al., 2008, 2009, 2011; Žarić et al., 2014). Readers with 4 years of reading experience showed an influence of letters presentation on the processing of speech sounds, but in a different temporal window than experienced adult readers. In beginner readers (with only 1 year of reading experience) or in DYS children, on the other hand, there was no indication of an early and automatic influence of (conflicting) letters during speech sound processing. These results suggest that beginner or DYS readers merely actively associate letters to speech sounds, whereas increasing experience with reading may lead to automatic LS integration (Froyen et al., 2008, 2009).

We propose that higher incongruency effect observed in left STC in beginning readers in the present study reflects this early stage of LS integration, which could reverse into congruency effect with increasing reading experience as observed in previous studies (Blau et al., 2009, 2010). The incongruency effect is also behaviorally relevant—in FHD− children or those who become typical readers the higher the incongruency effect in the left STS the better the performance in reading and reading-related tasks. Whereas FHD+ children show diminished incongruency effect, which is atypical for beginning readers. This result is in agreement with recent EEG-fMRI study examining audiovisual association processes of artificial stimuli in kindergarten children with familial risk for DYS (Karipidis et al., 2017). Higher familial risk for DYS correlated with diminished incongruency effect and children at a very high familial risk presented a congruency effect.

Conversely and rather unexpectedly, in the right hemispheric inferotemporal cortex, we found a congruency effect in FHD− children and an opposite effect (increased response to incongruent vs. congruent LS pairs) in the FHD+ group. This time the two groups differed only for the congruent condition. We did not find though any significant differences between children who later developed DYS and typical readers in brain response to neither congruent nor incongruent LS pairs in this region. Therefore, we suggest that the effects observed in the right inferotemporal cortex reflect early reading strategies based mostly on perceptual analysis of text and non-lexical form recognition system, which might be altered in FHD+ children. It was shown that in the course of reading acquisition, due to greater exposure to text, children shift from those strategies as reflected by progressive disengagement of the right ventral stream cortex (Turkeltaub et al., 2003).

In the current study additional FHD effects as well as early DYS predictors were found for unisensory conditions. When processing speech sounds, FHD+ children showed increased activation compared to FHD− group in right inferior and middle frontal gyri, possibly reflecting more effortful speech comprehension in at risk for DYS children (Monzalvo and Dehaene-Lambertz, 2013). Additional effects were found for the right aSTG ROI from Blau et al. (2010): children who developed DYS had higher activity in response to speech sounds than typically reading children. This result is in contrast to Blau et al. (2010) who found weaker activation to speech sounds in dyslexic compared to control children. It is in line though with studies on literate and illiterate subjects, where the response to speech in bilateral STG shows less activation for literate relative to illiterate participants (Dehaene et al., 2010).

Finally, for the unisensory letters, there was a trend for lower activity in the left fusiform gyrus ROI taken from Blau et al. (2010) in FHD+ compared to FHD− children. The location of this ROI is in close proximity to the visual word form area (VWFA) implicated in the processing of letters and words (Cohen et al., 2002; McCandliss et al., 2003; Cohen and Dehaene, 2004). Since the two groups had similar reading experience, it would be tempting to speculate that the abnormalities in the left occipitotemporal cortex in DYS (including hypoactivation in response to letters) is to some extent genetically driven and related to family risk. It is in agreement with anatomical studies showing less gray matter volume in left fusiform (Raschle et al., 2011) as well as atypical white matter organization in left ventral tract (Vandermosten et al., 2015) in FHD+ prereaders. Most importantly, however, we found no significant differences in the left fusiform between DYS and control children, even though behaviorally DYS children had lower letter knowledge and early reading skills. It is possible that differences between typical and DYS readers that emerge in orthographic processing later on may be a consequence of disordered crossmodal feedback into VWFA in DYS readers (Žarić et al., 2017), as differences in VWFA activity are not present in children at the start of formal reading education.

## Limitations

Even though we examined children with less than a year of formal reading education, only one third could be considered as prereaders. However, this relatively short period of formal reading instruction did not have a significant impact on the pattern of current findings. Additionally, we found a similar pattern of results in readers and prereaders (for details see Supplementary Figure S1 in Supplementary Materials), which supports the approach to pool these two groups in the current study. Yet, present results should be treated with caution until replicated on even younger, pre-reading children.

Retrospective selection of children who developed DYS resulted in largely unequal sample sizes (DYS = 17; TR = 58), which make heterogeneity of variance a problem. We thus performed bootstrap analyses to test for group differences on each measurement—performance on each behavioral test and brain activation in each ROI to specific stimuli (letters, speech sounds and congruent or incongruent LS pairs). In this way, it was not possible to test for interaction between multisensory (congruent and incongruent) conditions and group.

Moreover, this article was designed to follow the approach by Blau et al. (2010), thus in the main text we used identical statistical threshold for whole brain analyses, namely voxel-wise threshold of *p* < 0.01, corrected for multiple comparisons using cluster extent threshold of *p* < 0.05. Since nowadays such threshold might be regarded as liberal (Eklund et al., 2016), the analyses were repeated with a *p* < 0.005 voxel-wise threshold with an extent of 50 voxels common for pediatric studies (for e.g., Wang et al., 2018). Reassuringly all clusters reported in the main text survived this more stringent statistical approach (see Supplementary Table S1).

## Conclusion

Our study shows that alterations in brain activity during LS integration can be detected at very early stages of reading acquisition, suggesting their fundamental involvement in later reading impairments. Left STC actively responds to the conflicting LS pairs, which translates into better reading skills in children without the risk of developing DYS. The absence of such active response in FHD+ and even higher response to congruent LS in DYS in left PT could lead to failures in suppressing incongruent information during reading acquisition, which could result in future reading problems.

## Data Availability

The raw data supporting the conclusions of this manuscript will be made available by the authors, without undue reservation, to any qualified researcher.

## Author Contributions

JP analyzed and interpreted the data, drafted the manuscript. KC collected the data, analyzed and interpreted the data and revised the manuscript. ŁB helped with BrainVoyager software and revised the manuscript. MŁ, AD and AB collected the data and revised the manuscript. MW helped with data analysis. AM helped with fMRI design and revised the manuscript. NA helped with analysis and interpretation of the data and revised the manuscript. KJ designed the experiments, interpreted the data and drafted the manuscript. All the authors read and approved the final version of the manuscript.

## Conflict of Interest Statement

The authors declare that the research was conducted in the absence of any commercial or financial relationships that could be construed as a potential conflict of interest.

## Footnotes

^1^http://www.nitrc.org/projects/artifact_detect
