# Supplementary material for: Letter and Speech Sound Association in Emerging Readers With Familial Risk of Dyslexia
Source: Front Hum Neurosci. 2018 Oct 2;12:393. doi: 10.3389/fnhum.2018.00393 (PMC6176073; doi:10.3389/fnhum.2018.00393)
Supplement: Supplementary file 1 [file Table_1.DOCX]

**Supplementary materials**

**Analyses in readers vs. prereaders**

We split the group into prereaders (n=19, 12 FHD+/ 7 FHD-, 7 DYS/ 12 TR) and beginning readers (n=66; 38 FHD+/ 28 FHD-, 10 DYS/ 56 TR) and performed statistical tests on the ROIs taken from whole-brain analysis on the whole sample and from Blau et al. (2010). Prereaders could not read a single (two-letter) word in a minute, while readers read from 1 to 69 words per minute. For prereaders non-parametric U Mann-Whitney tests were used to compare demographics, behavioral performance and brain activation in ROIs to unisensory conditions in FHD+ and FHD- groups. To our knowledge, a non-parametric counterpart of two-way ANOVA is not available, so even though the groups are very small, especially FHD- (n=7) we performed a parametric two-way ANOVA to test for interaction between group and multisensory conditions. In case of readers, we used independent sample t-tests and a two-way ANOVA.

In both prereaders and readers there were no demographic or behavioral differences between FHD- and FHD+ children besides maternal (p=0.01 and p<0.001 for prereaders and readers) and paternal (p=0.044 and p<0.001 for prereaders and readers) ARHQ.

For prereaders, there was a trend for higher activation to speech sounds in FHD+ compared to FHD- children in the right MFG ROI from whole brain analysis (U=19, p=0.052). No other unisensory differences were significant.

Percent signal change in the group of prereaders for the multisensory ROIs is depicted in Figure S1 left panels. In the left PT/HS ROI (Blau et al., 2010) a significant group x condition interaction was found (F(1,17)=6.54, p=0.02). There was a trend for incongruent>congruent difference in FHD- group (p=0.051). In FHD+ differences were going into different direction, but did not reach significance. The group differences in each condition were not significant.

In beginning readers (see Figure S1, right panels), we found the same FHD effects in the clusters taken from whole brain analyses as in the total sample. In details, significant group x condition interaction was found in L PT/STG (F(1,64)=8.71, p=0.004), where groups differed for the incongruent condition (FHD->FHD+, p=0.023). In FHD- there was a trend for higher activation to incongruent>congruent (p=0.058) and reversed pattern in FHD+ (p=0.026). Interaction of group x condition was also significant in R ITG (F(1,64)=12.84, p=0.001), where groups differed for congruent condition FHD->FHD+ (p=0.009). In FHD- higher activation to congruent>incongruent was present (p=0.007) and a reversed pattern in FHD+ (p=0.028). Additionally in the left PT/HS ROI (Blau et al., 2010) there was a trend for significant group x condition interaction F(1,64)=3.19, p=0.079. Groups differed for the incongruent condition FHD->FHD+ p=0.016. In FHD+ there was a trend for congruent>incongruent (p=0.07), while in FHD- the difference between the conditions did not reach significance.

When we directly compared brain activation between pre-readers and readers using bootstrap analysis for all ROIs only in case of L STS in incongruent condition readers had higher activation than pre-readers (p=0.0095). No other between-group differences were observed. Overall, we argue that the pattern of results is very similar between pre-readers and beginning readers, while the observed differences might be a consequence of much smaller N in the prereading group, rather than reader vs. prereader status per se.


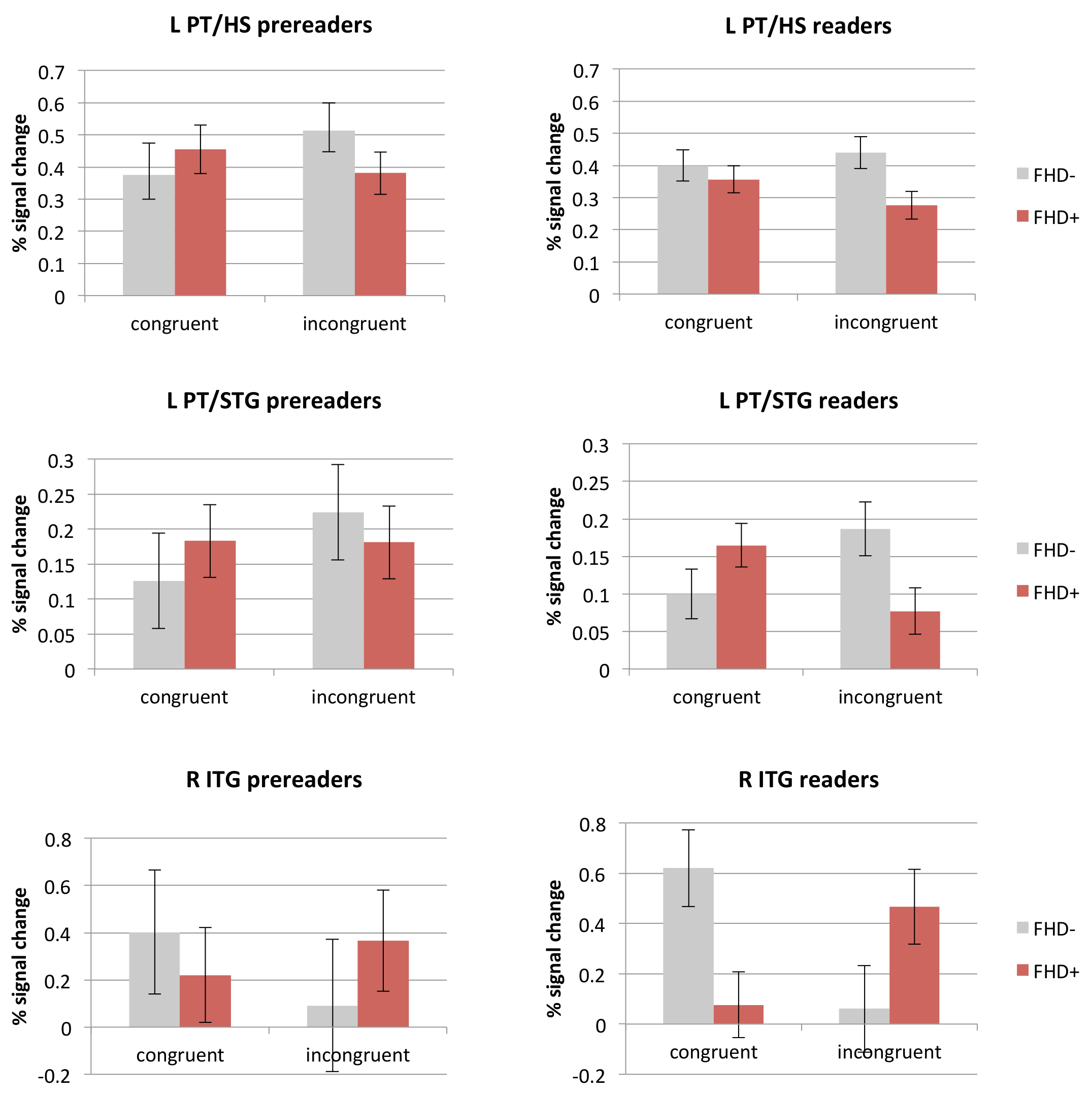


**Figure S1**. Bar plots showing % signal change for multisensory conditions (congruent and incongruent LS pairs) in prereaders (left panel) and readers (right panel) in three ROIs – left Planum Temporale/Heschl Sulcus (L PT/HS), left Planum Temporale/Superior Temporal Gyrus (L PT/STG) and right Inferior Temporal Gyrus (R ITG).

**Supplementary table 1**. Group differences in response to letters, speech sounds and for interaction between group and multisensory conditions at voxel-wise threshold of p < 0.005 and cluster extent of 50 voxels.

| **Brain region** | **BA** | **Hemisphere** | **x** | **y** | **z** | **t / F** | **Voxels** |
| --- | --- | --- | --- | --- | --- | --- | --- |
| **Letters: FHD- > FHD+** | | | | | | | |
| Fusiform gyrus | 19 | L | -35 | -71 | -17 | 3.39 | 71 |
| Middle temporal gyrus | 39 | R | 50 | -65 | 13 | 3.41 | 136 |
| **Letters: FHD+ > FHD-** |  |  |  |  |  |  |  |
| Inferior frontal gyrus | 45 | R | 62 | 24 | 6 | -3.78 | 128 |
| Inferior frontal gyrus | 47 | R | 58 | 32 | -4 | -3.82 | 130 |
| **Speech sounds: FHD+ > FHD-** |  |  |  |  |  |  |  |
| *Inferior Frontal Gyrus* | 10 | R | 54 | 28 | -1 | -4.11 | 549 |
| *Middle Frontal Gyrus* | 46 | R | 40 | 32 | 21 | -3.21 | 198 |
| Cerebellum (culmen) |  | L | -14 | -32 | -26 | -3.28 | 60 |
| Inferior frontal gyrus | 47 | L | -51 | 35 | -7 | -3.79 | 122 |
| **Congruency effect x FHD group interaction** | | | | | | | |
| *Inferior Temporal Gyrus* | 37 | R | 54 | -49 | -22 | 17.62 | 451 |
| *Planum Temporale, Superior temporal gyrus* | 41/13 | L | -41 | -21 | 19 | 13.77 | 645 |

Note: in italics are clusters reported in the main text at voxel-wise threshold of p < 0.01, corrected for multiple comparisons using cluster extent threshold of p < 0.05
